# DIAGNOSTIC VALUE OF C-REACTIVE PROTEIN AND THE INFLUENCE OF VISCERAL FAT IN PATIENTS WITH OBESITY AND ACUTE APPENDICITIS

**DOI:** 10.1590/0102-672020180001e1339

**Published:** 2018-03-01

**Authors:** Adham do Amaral e CASTRO, Thelma Larocca SKARE, Fernando Ide YAMAUCHI, Adriano TACHIBANA, Suheyla Pollyana Pereira RIBEIRO, Eduardo Kaiser Ururahy Nunes FONSECA, Andressa Tamy SAKUMA, Milena Rocha PEIXOTO, Mariana Athaniel Silva RODRIGUES, Maria Angela M. BARREIROS

**Affiliations:** 1 Imaging Department, Hospital Israelita Albert Einstein, São Paulo, SP; 2 Post-Graduate Program in Principles of Surgery, Evangelic Faculty of Paraná/University Evangelic Hospital of Curitiba/Medical Research Institute, Curitiba, PR, Brazil

**Keywords:** Apendicitis, Tomography, Obesity, Inflammation mediators., Apendicite, Tomografia, Obesidade, Mediadores da inflamação

## Abstract

**Background:**

The C reactive protein (CRP) is one of the most accurate inflammatory markers in acute appendicitis (AA). Obesity leads to a pro-inflammatory state with increased CRP, which may interfere with the interpretation of this laboratory test in AA.

**Aim:**

To assess sensitivity, specificity, positive predictive value (PPV), and negative predictive value (NPV) of CRP in patients with AA and their correlation to body mass index (BMI) and body fat composition.

**Method:**

This is a retrospective study based on clinical records and imaging studies of 191 subjects with histopathologically confirmed AA compared to 249 controls who underwent abdominal computed tomography (CT). Clinical and epidemiological data, BMI, and CRP values were extracted from medical records. CT scans were assessed for AA findings and body composition measurements.

**Results:**

CRP values increased according to patients’ BMI, with varying sensitivity from 79.78% in subjects with normal or lean BMI, 87.87% in overweight, and 93.5% in individuals with obesity. A similar pattern was observed for NPV: an increase with increasing BMI, 69.3% in individuals with normal or lean BMI, 84.3% in overweight, and 91.3% in individuals with obesity. There was a positive correlation between CRP and visceral fat area in patients with AA.

**Conclusions:**

Variations exist for sensitivity, specificity, PPV, and NPV values of CRP in patients with AA, stratified by BMI. An increase in visceral fat area is associated with elevated CRP across the BMI spectrum.

## RESUMO

**Racional:**

A proteína C reativa (PCR) é um dos marcadores inflamatórios com maior acurácia na apendicite aguda (AA). A obesidade leva a um estado pró-inflamatório com PCR aumentada, o que pode interferir na interpretação deste teste de laboratório na AA.

**Objetivo:**

Avaliar a sensibilidade, especificidade, valor preditivo positivo (VPP) e valor preditivo negativo (VPN) da PCR em pacientes com AA e sua correlação com o índice de massa corporal (IMC) e a composição da gordura corporal.

**Métodos:**

Este é um estudo retrospectivo baseado em registros clínicos e estudos de imagem de 191 indivíduos histopatologicamente confirmados com AA em comparação com 249 controles que foram submetidos à tomografia computadorizada abdominal (TC). Dados clínicos e epidemiológicos, valores de IMC e PCR foram extraídos de registros médicos. As TC foram avaliadas para achados de AA e medidas de composição corporal.

**Resultados:**

Os valores da PCR aumentaram de acordo com o IMC dos pacientes, com sensibilidade variável de 79,78% em indivíduos com IMC normal ou magro, 87,87% em excesso de peso e 93,5% em indivíduos com obesidade. Um padrão semelhante foi observado para o VPN: um aumento com o aumento do IMC, 69,3% em indivíduos com IMC normal ou magro, 84,3% em excesso de peso e 91,3% em indivíduos com obesidade. Houve uma correlação positiva entre a PCR e a área de gordura visceral em pacientes com AA.

**Conclusão:**

Existem variações quanto à sensibilidade, especificidade, VPP e valores VPN da PCR em pacientes com AA, estratificados pelo IMC. Um aumento na área de gordura visceral está associado à PCR elevada em todo o espectro do IMC.

## INTRODUCTION

Acute appendicitis (AA) is the second most common surgical emergency in the United States^11^. Diagnosis is essentially clinical, and laboratory tests are useful to guide surgical treatment decisions^1^.

 C-reactive protein (CRP) test is widely used to investigate AA, with a high positive likelihood ratio for diagnosis, especially when correlated to white blood cell count^3^. It is considered to be the inflammatory marker with highest diagnostic accuracy for AA with great negative predictive values (NPV)^16^. CRP is also a useful biomarker to assess drug treatment response^14^ and to identify cases with potential for clinical complications^20^.

 Despite the exposure to ionizing radiation, computed tomography (CT) is the most accurate imaging modality for AA diagnosis^21^. AA-specific CT diagnostic criteria may include: intraluminal appendicolith, absence of intraluminal air, parietal contrast enhancement, parietal thickening, periapendicular fat densification, periapendicular fluid and lymphadenopathy in the lower right abdominal quadrant^23^. Highly suspected cases can be securely diagnosed by clinical examination performed by an experienced surgeon, without using CT. However, when the clinical presentation involves only few diagnostic criteria, imaging serves as a critical aid in the diagnostic approach^23^.

Patients with higher body mass index (BMI) and abnormal lipid profiles may be at risk for a pro-inflammatory and chronic prothrombotic state^15^. Among abnormal serum parameters specific to obesity, CRP is a biomarker that becomes elevated in such patients, making its interpretation sometimes difficult in associated inflammatory conditions as AA^7^ ^,^ ^10^ ^,^ ^15^.

Since CRP can serve as an important diagnostic tool in ultimately informing the decision for AA surgery, and since its value can be subject to modification by body fat content, the present study aimed to evaluate CRP sensitivity, specificity, positive predictive value (PPV), and negative predictive value (NPV) in AA diagnosis according to BMI in subjects with histopathologically confirmed AA diagnosed by CT. A secondary endpoint was to evaluate the impact of fat compartments (visceral, subcutaneous or intramuscular) in CRP modifications.

## METHOD

This is an observational, retrospective, case-control study. The study was approved by the Institutional Board Review and Ethics Committee in Research, with a waiver for informed consent due to its retrospective design.

### Subject selection

#### *Inclusion criteria*

Were reviewed 286 abdominal and pelvic CT scans performed in patients with subsequent histopathologically confirmed AA performed from January 1, 2014 to December 31, 2014. Medical records and laboratory results for each subject were reviewed. Inclusion criteria included that each patient had to receive AA diagnosis from a CT scan with subsequent surgical treatment and confirmation by histopathological review.

#### *Exclusion criteria*

The following criteria were adopted as exclusion criteria: age under 18 years old, tomographic or histopathological diagnosis other than AA, non surgical treatment, and no CRP test ordered during medical care. Patients below 18 years old were excluded since BMI analysis patterns could not be equally applied to them as compared to adults, which would limit analyses and data interpretation.

### Study sample

#### *Cases*

Out of 286 patients with AA CT diagnosis, 95 were excluded due to the following reasons: 65 subjects did not undergo appendectomy, receiving only subsequent clinical treatment; and 30 subjects did not have an available CRP test. Therefore, after inclusion and exclusion criteria, the case group was comprised of 191 subjects.

#### *Controls*

The control group was formed by subjects that underwent abdominal and pelvic CT exams for abdominal pain in the same period, with normal imaging results, comprising 249 patients matched by age, gender, and BMI to the case group. With the exception of CT and histopathological diagnosis of AA and surgical procedure, the same inclusion and exclusion criteria were applied to the control group.

### Clinical Information

#### *Medical and laboratory records*

Medical records were assessed for demographic data (gender, weight, height, and BMI), clinical and laboratory parameters, and Alvarado score. BMI results between 18.5 and 24.9 kg/m^2^ were classified as normal, between 25 and 29.9 kg/m^2^ as overweight, and equal to or higher than 30 kg/m^2^ as obese. The latter group was subgrouped as Class 1 obesity if BMI values were between 30 and 34.9 kg/m^2^; Class 2 obesity if between 35 and 39.9 kg/m^2^; and Class 3 obesity if values equal to or higher than 40 kg/m^2^. BMI values lower than 18.5 kg/m^2^ were considered lean (underweight)^9^.

CRP values were tabulated for each subject. This test is obtained in our hospital through an immunoturbidimetric assay Ortho Vitros Fusion 5.1 FS (Ortho Clinical Diagnostics, Rochester, NY), considering results lower than 5 mg/l as normal.

 Patients’ white blood cell count was also noted, with normal absolute count ranging from 3,500 to 10,500 ul and neutrophils from 1,700 to 8,000 ul. This electronic count is performed using XS-1000i equipment.

#### *Imaging exams*

All scans were performed using the Toshiba Aquilion 64-slice MDCT scanner (Toshiba America Medical Systems, Inc., Tustin, CA). The following tomographic criteria were considered for the diagnosis of AA: intralumial appendicolith, absence of intraluminal air, parietal contrast enhancement, parietal thickening, periapendicular fat densification, periapendicular fluid and lymphadenopathy in the lower right abdominal quadrant. Additional findings were also added to the data collection spreadsheet.

An estimation of body composition was made using sliceOmatic® software (TomoVision, Montreal, CA), based on tomographic slices to separate fat content (which includes subcutaneous, visceral fat, and intramuscular fat) from muscle mass, the latter demonstrated by muscular tissue, separated by tomographic attenuation means based on an axial image at the level of third lumbar vertebrae^13^. Tissues were classified according to Hounsfield tomographic units from -29 to 150 as musculature, -190 to -30 as subcutaneous fat and intramuscular fat, and -50 to -150 as visceral fat^13^. To estimate total body composition (i.e, total body fat and muscle weight in kg), were used mathematical formulas that demonstrated good precision for tomographic evaluation when compared to whole-body dual-energy X-ray absorptiometric method, a lesser available method but considered as the gold-standard^13^. Conversion formulas used were^13^:

Total fat mass (kg)=0.042 [sum of visceral, intramuscular and subcutaneous fat, at L3 level using CT (cm^2^)] + 11.2;

Total lean mass (kg)=0.14 [muscular tissue at L3 level using CT (cm^2^)] + 0.72.

**FIGURE 1:**
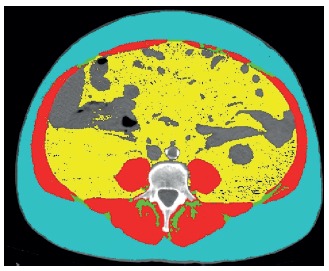
Image example of CT on l3 level axial plane, analyzed with sliceomatic®: the red color indicates the skeletal muscle; green indicates the intramuscular adipose tissue; yellow the visceral adipose tissue and the blue the subcutaneous adipose tissue

Software sliceOmatic® was used for patients that showed appendicitis, extracting the amount of fat and lean mass (in cm^2^), that was subsequently converted to total fat and lean mass by the formulas described above. Figure 1 represents an axial CT image analyzed using sliceOmatic®.

### Statistical analyses

Data were tabulated in frequency and contingency tables. Normal distribution of the sample was analyzed using Kolmogorov-Smirnov test. Measures for central tendency were expressed as means and standard deviation for Gaussian and as median and interquartile ranges (IQR) for non-Gaussian distribution. Nominal data association tests were performed using Fisher and chi-square tests; numerical data by Mann Whitney (non-Gaussian) and unpaired t-test (Gaussian). Correlation analysis of serum CRP values with different types of body fat was performed by Spearman test. Calculations were made using Graph Pad Prism® software version 5.0. Significance level was set at 5%. Specificity, sensitivity, and PPV and NPV of CRP in each BMI group (normal / lean, overweight and people with obesity) were calculated.

## RESULTS

### Sample description and matched data

Cases (191 subjects) and controls (249 subjects) were matched for age, gender, and BMI. 

### CRP, clinical, laboratory and CT findings evaluation according to BMI

**FIGURE 2:**
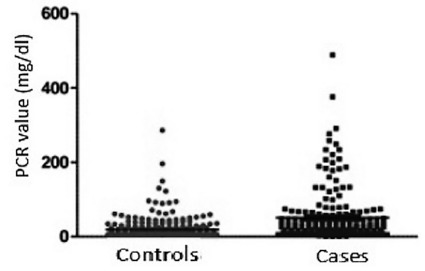
Comparison of PCR values in AA cases and controls

 As expected, CRP measured values were higher in the case group (Figure 2).

PCR values in AA cases=0.30-489.0 mg/dl (median of 23.0; IQR of 8.2-57.0); Controls=5.0-286.0 mg/dl (median of 5.0; IQR of 5.0-19.6) with p<0.0001 (Mann Whitney).

Table 1 shows the comparison values in the case group between subjects with lean/normal BMI and subjects with overweight BMI regarding clinical, laboratory and CT data. Clinical and CT data did not differ in this subset.

In Table 2 the comparison is made between lean/normal BMI individuals and those with obesity. Differences in the number of patients with increased CRP are observed, as well as a tendency for higher CRP values in subjects with obesity over lean/normal BMI. Subjects with obesity have a 5.6-fold higher chance to present with increased CRP than subjects with lean/normal BMI. Furthermore, individuals with obesity are 2.6 times more likely to have pain and defense on palpation in the lower right quadrant, and 4.8 times more likely to have fever. Was observed that CT findings do not differ from people with lean/normal BMI and people with obesity (not showing an increased number of complicated cases in these subjects that justify the abnormalities found).

**TABLE 1:** Comparative data in AA between lean and normal BMI individuals with overweight BMI individuals

	Lean and normal n=94	Overweight n=66	p
BMI (kg/m2) (||)	17.5-24.9 Median of 23.1(21.4-24.3)	25.22-29.94 Median of 27.04 (25.9-28.1)	<0.0001(*)
Migrating pain	9/94 - 9.5%	7/66- 10.6%	0.83 (*†*)
Anorexia	5/94 - 5.3%	5/66 - 7.5%	0.56 (*†*)
Nausea and vomiting	37/94 - 39.3%	32/66 - 48.4%	0.25 (*†*)
Pain and defense in the right lower quadrant	42/94 - 44.6%	27/66-40.9%	0.63 (*†*)
Blumberg’s Pain	10/94 - 10.3%	4/66-6.06%	0.31 (*†*)
Fever	11/94 - 11.3%	8/66 - 12.1%	0.93 (*†*)
Leukocytosis	67/94 - 71.2%	48/56- 85.7%	0.84 (*†)*
Left shift	26/94-27.6%	11/66-16.6%	0.10 (*†*)
Leukocyte value	4.560-26.130 Mean of 13.280±4.571	5.720-19.860 Mean of 12.830±3.751	0.51 (*‡*)
Number of patients with increased CRP	75/94- 79.7%	58/66-87.8%	0.17(*)
CRP value (mg/dL) (¶)	0.3-489 Median of 21.5(8.1-50.2)	2.8-376.2 Median of 17.0 (7.6-47.0)	0.60 (*)
Alvarado score	0-7 Median of 3.0 (2.0-4.0)	0.0-8.0 Median of 3.0(2.0-4.0)	0.97 (*)
Tomographic findings			
Intraluminal appendectomy	24/94- 25.5%	2/66-18.1%	0.27 (*†*)
Absence of intraluminal air	69/94- 73.4%	41/66- 62.1%	0.12 (*†*)
Parietal enhancement by contrast	66/94- 70.2%	37/66- 56.06%	0.06 (*†*)
Parietal thickening	91/94- 96.8%	64/66-96.9%	1.00 (*§*)
Densification of appendicular fat	84/94 - 89.3%	64/66- 96.97%	0.12 (*§*)
Periappendicular fluid	25/94- 25.5%	15/66-22.7%	0.57(*†*)
Lymphadenopathy	18/94- 19.1%	9/66-13.6%	0.35 (*†*)

(*)=Mann Whitney test; (†)= chi-square test; (‡) =unpaired t-test; (§) =Fisher’s test; (||) BMI=body mass index; (¶) CRP=C-reactive protein

### Sensitivity, specificity, PPV, and NPV of CRP in AA according to BMI

**FIGURE 3:**
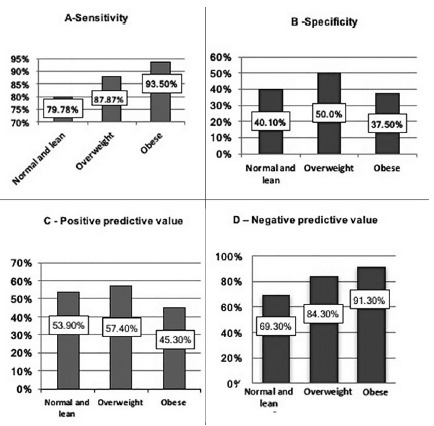
CRP sensitivity, specificity, PPV and NPV in AA, according to patient BMI

Sensitivity, specificity, PPV and NPV are presented in Figure 3. In subjects with obesity, CRP sensitivity increases along with increased BMI, while lower specificity is seen in these subjects. Was observed a decrease in CRP PPV and increase in NPV according to increase in BMI.

**TABLE 2:** Comparative data in AA between lean and normal BMI individuals with individuals with obesity

	Lean and normal n=94	Individual with obesity n= 31	p
BMI (kg/m2) (¶)	17.5-24.9 Median of 23.1(21.4-24.3)	30.07-39.35 Median of 32.69 (31.1-36.1)	<0.0001 (*)
Migrating pain	9/94 - 9.5%	4/31	0.73 (†)
Anorexia	5/94 - 5.3%	2/31	1.00 (†)
Nausea and vomiting	37/94 - 39.3%	16/31	0.23 (‡)
Pain and defense in the right lower quadrant	42/94 - 44.6%	21/31- 67.7%	**0.02** (‡) (a)
Blumberg’s Pain	10/94 - 10.3%	5/31- 16.12%	0.52 (†)
Fever	11/94 - 11.3%	12/31- 38.7%	0.0007 (‡) (b)
Leukocytosis	67/94 - 71.2%	19/31-61.29%	0.29 (‡)
Left shift	26/94-27.6%	6/31- 19.35%	0.35(‡)
Leukocyte value	4.560-26.130 Mean of 13.280±4.571	6,600-21,170 Mean of 12.510±3.739	0.39 (||)
Number of patients with increased CRP	75/94- 79.7%	29/31 - 93.55%	**0.01**(‡)(c)
CRP value (mg/dL) (#)	0.3-489 Median of 21.0(8.1-50.2)	5.0-258.0 Median of 31.0 (16.0-99.0)	0.07(*)
Alvarado score	0-7 Median of 3.0 (2.0-4.0)	0-7 Median of 3.0(2.0-5.0)	0.23 (*)
Tomographic findings			
Intraluminal appendicolith	24/94- 25.5%	4/31 - 12.9%	0.21 (†)
Absence of intraluminal air	69/94- 73.4%	27/31 - 87.09%	0.14 (†)
Parietal enhancement by contrast	66/94- 70.2%	19/31 - 61.29%	0.35 (‡)
Parietal thickening	91/94- 96.8%	31/31 - 100%	0.57 (†)
Densification of appendicular fat	84/94 - 89.3%	29/31 - 93.55%	0.72 (‡)
Periappendicular fluid	25/94- 25.5%	6/31 - 19.35%	0.41 (‡)
Lymphadenopathy	18/94- 19.1%	8/31 - 25.8%	0.42 (‡)

(*)=Mann Whitney test; (†)=Fisher’s test; (‡)=Chi-square test; (||)=unpaired t-test. (¶) BMI=body mass index; (#) CRP=C-reactive protein; (**) OR=odds ratio; (††) CI=confidence interval. (a) OR=2.6; 95% CI 1.10-6.12; (b) OR=4.82; 95% CI=1.85-12.57; (c) -OR=5.84; 95% CI=1.30-26.22

BMI=body mass index; normal and lean BMI=under 24.9 kg/m^2^; overweight=BMI of 25 to 29.9 kg/m^2^; obesity=BMI>30 kg/m^2^

### Body fat distribution and correlation of serum PCR values

From a total of 191 subjects, 146 had their body fat composition analyzed by CT. From this subgroup, 45 patients were excluded due to suboptimal acquision protocol leading to reduced equipment “field of view”, a technical parameter that prevented SliceOmatic*®* software analyses. 

Table 3 shows descriptive profile of the parameters evaluated, and Table 4 shows correlation of CRP values with body fat composition in all subjects, not demonstrating significant differences.

**TABLE 3:** Descriptive study of variables studied by imaging (n=146) for muscular mass data, subcutaneous fat, visceral, intramuscular, total fat mass and fat-free mass

	Muscle (cm²)	Intramuscular fat (cm²)	Visceral fat (cm²)	Subcutaneous fat (cm²)	Fat Mass (Kg)	Fat-free mass (Kg)
Minimum value	50.00	0.8281	1.520	48.77	11.20	0.72
IQR 25% (*)	113.5	5.000	33.32	160.4	14.90	13.99
Median	137.9	8.392	67.20	214.8	21.23	17.76
IQR 75%(*)	176.1	13.46	140.5	273.9	26.54	23.43
Maximum value	242.1	51.72	421.1	546.9	45.74	34.61

(*)IQR=interquartile range; CRP=C-reactive protein

**TABLE 4:** CRP correlation study (mg/dl) with body composition data (Spearman test)

	Spearman Rho	95% CI	p
Muscle (cm²)	0.03	-0.13 - 0.19	0.71
Intramuscular fat (cm²)	-0.10	-0.26 - 0.06	0.20
Visceral fat (cm²)	-0.06	-0.22 - 0.10	0.44
Subcutaneous fat (cm²)	-0.10	-0.26 - 0.06	0.20
Fat mass (kg)	-0.10	-0.24 - 0.041	0.14
Fat-free mass (kg)	-0.02	-0.17 - 0.11	0.70

(*)CI=confidence interval

Finally, CRP values were evaluated according to each body composition type data considering the values of subjects above and below the median value in each measurement (muscle, intramuscular fat, visceral fat, subcutaneous fat, total fat tissue and fat-free tissue). These data analyses revealed significant differences for subjects with visceral fat values above the median, as seen in Table 5.

**TABLE 5:** Correlation study of serum CRP (mg/dl) with body fat distribution variables according to median value (Spearman test)

	Above median values		Below median values		
Variable	Rho	**95% CI** (*)	p	Rho	95%CI
Muscle (cm²)	0.03	-0.20 - 0.26	0.78	-0.06	-0.29 - 0.17
Intramuscular fat (cm²)	0.07	0.01 - 0.43	0.05	0.07	-0.16 - 0.30
Visceral fat (cm²)	0.25	0.02 - 0.46	0.02	-0.01	-0.24 - 0.22
Subcutaneous fat (cm²)	0.04	-0.19 - 0.27	0.70	0.06	-0.16 - 0.30
Fat mass (kg)	0.10	-0.098 - 0.31	0.28	-0.08	- 0.28 - 0.12
Fat-free mass (kg)	-0.05	-0.26 - 0.14	0.56	0.03	-0.17 - 0.23

(*)CI=confidence interval

## DISCUSSION

CRP is considered to be one of the serum inflammatory markers with higher diagnostic accuracy in patients with AA^3^. Therefore, this study focused on CRP measurement in patients with AA and controls, as well as the correlation of its value with different body fat compartments. As expected, CRP values were higher in AA patients when compared to controls.

 The Alvarado score is based on signs, symptoms and laboratory data for diagnosis of AA^2^. It is known to be useful in the AA clinical condidition^2^, especially if associated to CRP results^22^, but it did not show differences for subjects with AA when correlated to BMI in this study. We believe that this finding is related to the fact that there are not many clinical differences in patients with AA who have obesity, since this tool uses various clinical data for its calculation. However, univariate analysis reveal that subjects with increased weight tend to present with more fever (38% vs. 11%), and more pain and defense in the right lower quadrant (67% vs. 44%), compared to those subjects with lean/normal BMI. This result may be attributed to higher inflammation level and pain perception alteration secondary to obesity^8^ ^,^ ^24^ ^,^ ^25^. Obesity also seems to be related to leukocytosis^17^ ^,^ ^19^, as confirmed in the present study.

These data indicate that the evaluation of pain and interpretation of laboratory parameters in patients with obesity should be made with care, avoiding misdiagnosis, since obesity can act as a confounding factor for clinical and laboratory results.

CT findings related to AA diagnosis in the present sample converge with the literature data, and are therefore not likely affected by obesity, including: parietal thickening (186/191 - 97.3%), periapendicular fat densification (177/191 - 92.6%), absence of intraluminal air in the appendix (137/191 - 71.72%) and parietal contrast enhancement (123/191 - 64.3%)^23^.

 The most significant finding of the current study was the changes in sensitivity, specificity, and consequently PPV and NPV of CRP in AA diagnosis, which corresponded to the main objective of this study. Higher sensitivity was observed with increasing BMI (91.3% in subjects with obesity, 84.3% in overweight BMI, and 69.3% in lean/normal BMI subjects); however, this increase in sensitivity is of course at the expense of specificity test loss. This finding is understandable, since, as discussed above, CRP is increased at baseline levels in individuals with obesity, even without an appendicitis diagnosis. However, surgeon’s questions regarding the patient with abdominal pain, such as “Is this appendicitis or not?”, or “Should I operate or not?”, are better supported by stronger specificity. Stronger specificity will show that a normal test suggests against AA diagnosis, or, seen differently, how much of the test change actually is due to AA, despite obesity as a confounding variable. Therefore, high CRP values in subjects with obesity should be less appreciated than in a normal BMI subject, due to the risk to incur in a false positive case.

 Sensitivity and specificity findings in this study are also in agreement with available literature, highlighting that these values should be interpreted with care, in order to avoid unnecessary surgical interventions^10^.

Fat tissue is considered today as a multifunctional organ, far beyond its traditional energy storage function. It has an important role in pro-inflammatory molecule release, such as Interleukin-6 (IL-6), tumor necrosis factor (TNF) alpha and leptin, which act on both local and systemic inflammation conditions^12^. Some patients with obesity suffer from a systemic pro-inflammatory state called adiposopathy, which is associated with the increase for developing multiple diseases^4^ ^,^ ^27^. In the present study, the increase of CRP among patients with AA was seen in the subset of individuals with obesity when correlated to normal or lean BMI.

The present study also evaluated body composition of patients with AA by sliceOmatic® software and subsequent conversion by means of mathematical formulas to estimate body muscular and fat composition. Such analysis is of major importance, since body fat distribution, is more associated to harmful obesity consequences, than total fat area, specifically in that visceral fat is most associated with adiposopathy^26^.

Specific mechanistic reasons for why visceral fat may cause inflammatory damage remain controversial. Adipocytes of visceral fat were thought to mediate insulin resistance through secretion of free fatty acids and adipokines^5^. However, other factors could explain why visceral fat is responsible for inflammatory response increases: its localization, allowing the entrance of metabolically active substance directly into the liver through the portal system, and because this fat depot has particular pharmacokinetic characteristics allowing for secreted molecules to spread easily through the body ^5^. 

Data from our study demonstrate that in subjects with greater visceral fat area there is an influence on CRP values, in agreement with the concept of adiposopathy and the prominent role of visceral fat in this condition. These findings are related to the role of obesity, notably visceral fat, in the contribution of a higher degree of inflammation in patients with AA. To our knowledge, this is the first study to analyze visceral obesity’s contribution to pro-inflammatory state in subjects with AA.

 This study had several limitations. First, is its retrospective design. Although were excluded surgical cases due to other acute abdomen pathologies that could increase CRP levels, individuals may have presented with different inflammatory conditions that may have been able to raise CRP levels in a normal abdominal CT. Future studies are encouraged using a prospective design, and allowing control group characterization without any clinically detectable inflammatory conditions. 

 Results observed in the present study indicate how obesity, especially due to visceral fat, is capable of impacting the interpretation of clinical data and laboratory tests. This association may interfere in diagnosis rationale and surgical decisions. As future perspectives, considering the global increase in the prevalence of obesity, especially among subjects presenting with AA, body fat compositions routinely provided by CT and magnetic resonance imaging radiological reports could improve clinical and surgical approaches in subjects with suspected AA. 

## CONCLUSION

Changes in CRP sensitivity, specificity, PPV, and NPV values in patients with AA were seen according to BMI, especially an increase in sensitivity due to a decrease in specificity with BMI increase. Only visceral fat correlated to CRP values.

## References

[1] Al-Abed YA, Alobaid N, Myint F (2015). Diagnostic markers in acute appendicitis. Am J Surg.

[2] Alvarado A (1986). A practical score for the early diagnosis of acute
appendicitis. Ann Emerg Med.

[3] Andersson REB (2004). Meta-analysis of the clinical and laboratory diagnosis of
appendicitis. Br J Surg.

[4] Bays H (2014). Adiposopathy, "sick fat," Ockham's razor, and resolution of the
obesity paradox. Curr Atheroscler Rep.

[5] Bergman RN, Kim SP, Catalano KJ, Hsu IR, Chiu JD, Kabir M (2006). Why visceral fat is bad mechanisms of the metabolic
syndrome. Obesity (Silver Spring).

[6] Bliss LA, Yang CJ, Kent TS, Ng SC, Critchlow JF, Tseng JF (2015). Appendicitis in the modern era universal problem and variable
treatment. Surg Endosc.

[7] Bonomini F, Rodella LF, Rezzani R (2015). Metabolic syndrome, aging and involvement of oxidative
stress. Aging Dis.

[8] D'arcy Y (2012). Pain and obesity. Nurs Manag.

[9] (1992). Gastrointestinal surgery for morbid obesity: National Institutes
of Health Consensus Development Conference Statement. Am J Clin Nutr.

[10] Kutasy B, Laxamanadass G, Puri P (2010). Is C-reactive protein a reliable test for suspected appendicitis
in extremely obese children. Pediatr Surg Int.

[11] Masoomi H, Mills S, Dolich MO, Ketana N, Carmichael JC, Nguyen NT (2011). Comparison of outcomes of laparoscopic versus open appendectomy
in adults data from the Nationwide Inpatient Sample (NIS),
2006-2008. J Gastrointest Surg.

[12] Moulin CM, Marguti I, Peron JPS, Rizzo LV, Halpem A (2009). Impact of adiposity on immunological parameters. Arq Bras Endocrinol Metab.

[13] Mourtzakis M, Prado CM, Lieffers JR, Reiman T, Mccargar LJ, Baracos VE (2008). A practical and precise approach to quantification of body
composition in cancer patients using computed tomography images acquired
during routine care. Appl Physiol Nutr Metab.

[14] Okus A, Ay S, Karahan Ö, Eryilmaz MA, Sevinç B, Aksoy N (2015). Monitoring C-reactive protein levels during medical management of
acute appendicitis to predict the need for surgery. Surg Today.

[15] Orenes-Piñero E, Pineda J, Roldán V, Hernández-Romero D, Marco P, Tello-Montoliu A (2015). Effects of Body Mass Index on the Lipid Profile and Biomarkers of
Inflammation and a Fibrinolytic and Prothrombotic State. J Atheroscler Thromb.

[16] Panagiotopoulou IG, Parashar D, Lin R, Antonowicz S, Wells AD, Bajwa FM (2013). The diagnostic value of white cell count, C-reactive protein and
bilirubin in acute appendicitis and its complications. Ann R Coll Surg Engl.

[17] Papalou O, Livadas S, Karachalios A, Tolia N, Kokkoris P, Tripolitakis K (2015). White blood cells levels and PCOS direct and indirect
relationship with obesity and insulin resistance, but not with
hyperandogenemia. Hormones (Athens).

[18] Perez Rodrigo C (2013). Current mapping of obesity. Nutr Hosp.

[19] Riley LK, Rupert J (2015). Evaluation of patients with leukocytosis. Am Fam Physician.

[20] Sammalkorpi HE, Leppäniemi A, Mentula P (2015). High admission C-reactive protein level and longer in-hospital
delay to surgery are associated with increased risk of complicated
appendicitis. Langenbecks Arch Surg.

[21] Shogilev DJ, Duus N, Odom SR, Shapiro NI (2014). Diagnosing appendicitis evidence-based review of the diagnostic
approach in 2014. West J Emerg Med.

[22] Thirumallai S, Wijesuriya SR, Mitchell A, Delriviere L (2014). Predictive value of C-reactive protein with Alvarado score in
acute appendicitis. ANZ J Surg.

[23] Thompson AC, Olcott EW, Poullos PD, Jeffrey RB, Thompson MO, Rosenberg J (2015). Predictors of appendicitis on computed tomography among cases
with borderline appendix size. Emerg Radiol.

[24] Torensma B, Thomassen I, Van Velzen M, In't Veld BA (2016). Pain experience and perception in the obese subject systematic
review (revised version). Obes Surg.

[25] Tramullas M, Finger BC, Dinan TG, Cryan JF (2016). Obesity Takes Its Toll on Visceral Pain High-Fat Diet Induces
Toll-Like Receptor 4-Dependent Visceral Hypersensitivity. PLoS One.

[26] Wu CK, Yang CY, Lin JW, Hsieh HJ, Chiu FC, Chen JJ (2012). The relationship among central obesity, systemic inflammation,
and left ventricular diastolic dysfunction as determined by structural
equation modeling. Obesity (Silver Spring).

[27] Yamauchi FI, Castro ADAE (2017). Obesity, adiposopathy, and quantitative imaging
biomarkers. Radiol Bras.

